# Multi-year dataset on daily electricity demand, generation, load shedding, and external conditions in Bangladesh

**DOI:** 10.1016/j.dib.2025.112014

**Published:** 2025-08-29

**Authors:** Md. Ikrama Hossain, Tasnia Nafs, Sakif Yeaser, Asif Newaz

**Affiliations:** Department of Electrical and Electronic Engineering, Islamic University of Technology, Gazipur 1704, Bangladesh

**Keywords:** Load forecasting, Energy policy, Seasonality, Time series, Long-term prediction

## Abstract

This dataset compiles daily electricity statistics for Bangladesh across national and divisional levels. The data were programmatically scraped from the Bangladesh Power Development Board's (BPDB) digital archive and processed into a structured, machine-readable format using a custom Python pipeline. The dataset consists of 1867 daily reports, spanning from November 21, 2019, to December 30, 2024. Each record includes key variables such as electricity demand, generation, load shedding, temperature, and supply limitations due to gas shortages, coal availability, and low water levels. The dataset was curated through multiple stages, which include manual verification, holiday classification, missing value imputation, and outlier correction. Five sequential versions are provided, which reflect progressive enhancements from raw extraction to modeling readiness. The data can be used in time series analysis, load forecasting, energy policy research, and machine learning applications in resource-constrained settings. Additionally, the collection spans the COVID-19 pandemic period, offering unique opportunities for studying the impact of external factors on national energy systems.

Specifications Table

**Table:** 

Subject	Engineering & Materials science
Specific subject area	Electricity generation, demand and power generation constraint-related data for Bangladesh
Type of data	Table
Data collection	The dataset was scraped from https://misc.bpdb.gov.bd/daily-generation-archive using a custom Python-based automation pipeline. It includes daily reports from 21 November 2019 to 30 December 2024, covering national and divisional electricity demand, forecasts, generation data, and supply limitations. Each PDF report was downloaded from the BPDB website, parsed, and tabulated. Afterward, the data was systematically compiled into Excel files, followed by rigorous cleaning and preprocessing to ensure accuracy and consistency before producing the final dataset. For transparency and reproducibility, the complete Python pipeline is openly available at the GitHub repository (Bangladesh-Power-Data-Pipeline).
Data source location	The dataset was collected from Bangladesh, covering the national level and nine regions: Dhaka, Chattogram, Rajshahi, Khulna, Rangpur, Mymensingh, Sylhet, Barishal and Cumilla. The geographical location of Bangladesh is approximately 23°48′N latitude and 90°24′E longitude.
Data accessibility	Repository name: Structured Dataset of Daily Electricity Demand, Generation, Load Shedding, and Supply Constraints in Bangladesh (2019–2024). Data identification number: 10.17632/x7r7wdb39k.1 Direct URL to data: https://data.mendeley.com/datasets/x7r7wdb39k/1 [1]
Related research article	S. Yeaser, T. Nafs, Md.I. Hossain, Comparative Analysis of Deep Learning Models for Long-Term Electricity Demand Forecasting in Bangladesh Using Web-Scraped Data, in: 2025 17th International Conference on Electronics, Computers and Artificial Intelligence (ECAI), IEEE, Târgoviște, 2025: pp. 1–7. https://doi.org/10.1109/ECAI65401.2025.11095479 [2].

## 1 Value of the Data

• Collected over several years, this dataset records the day-to-day dynamics of Bangladesh’s electricity sector, including demand, generation patterns, load shedding, and associated constraints at national and divisional levels.
• Besides time series forecasting, the dataset supports a wide range of applications, including anomaly detection, load-shedding pattern analysis, peak demand estimation, generation-consumption gap evaluation, and the development of energy management systems, offering a comprehensive view of the country’s energy demand and supply.
• The dataset can be used to investigate demand variability, model stress scenarios on the power grid, or evaluate the influence of temperature, holidays, and fuel availability on energy dynamics.
• The structured format and engineered features make the data suitable for both statistical modelling and machine learning (ML) applications, including classification, clustering, regression, and causal inference studies.
• As this dataset includes data from the beginning to the end of the COVID-19 pandemic, it provides valuable insights into the impact of a global pandemic on energy management in a developing country like Bangladesh.
• The dataset is accompanied by a reproducible data collection and preprocessing pipeline, enabling others to update, extend, or adapt the dataset to similar regional contexts or new research questions.

## 2 Background

This dataset is compiled to facilitate long-term forecasting of daily electricity demand and load shedding across Bangladesh at both national and divisional scales. It is designed to support the application of advanced recurrent neural network architectures commonly used in multivariate time series forecasting.

The data is sourced from 1867 daily reports published by the Bangladesh Power Development Board (BPDB), which were collected through a scripted process using a Python-based automation framework incorporating BeautifulSoup, pdfplumber, and pandas. The extracted tabular information underwent rigorous preprocessing, including the imputation of missing values using statistical methods, outlier smoothing, and the construction of auxiliary features. These features include temporal attributes such as weekday and month annotations, holiday classifications, and temperature data.

The resulting dataset was utilized in the conference paper titled “Comparative Analysis of Deep Learning Models for Long-Term Electricity Demand Forecasting in Bangladesh Using Web-Scraped Data”, presented at ECAI 2025 (https://ecai.ro). By publishing the dataset in a standardized and reusable format, this article enhances transparency and promotes further research in data-driven energy forecasting and planning in resource-constrained contexts.

## 3 Data Description

The repository contains five sequential versions of a daily electricity demand and generation dataset, progressing from raw extraction to a finalized, analysis-ready form. All versions are provided as single-sheet Excel files, stored within subfolders. Each dataset contains daily entries beginning on November 21, 2019, with each version building on the previous through cleaning, feature engineering, and modeling preparation. However, a total of 17 dates are currently missing from the dataset, as the corresponding PDFs were not found on the official site. These missing dates include January 2, March 27, and March 28 of 2020; January 23, March 15, April 1, May 8, May 16, and July 5 of 2023; and June 3, July 1, and July 18 to July 23 of 2024.

### 3.1 Version 1: Raw Extract

The initial version comprises the unprocessed dataset extracted directly from daily BPDB reports. It is provided as a single Excel file with one worksheet containing 41 columns, each aligned row-wise without merged headers or multi-level indexing. Spanning from November 21, 2019, to December 30, 2024, each row represents a calendar day. The dataset includes both numerical and textual data, retaining all inconsistencies, missing values, and zero entries as they appeared in the source files. No transformations, scaling, or standardizations were applied, preserving the raw format for full traceability.

**Fig. 1:**
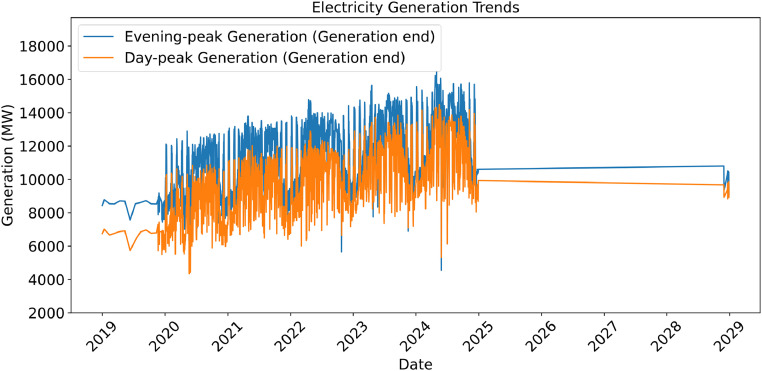
Daily Trends of Peak Electricity Generation (2019–2024) - Raw Extracted Data.

Fig. 1 illustrates daily trends in evening and day peak electricity generation, revealing seasonal and annual patterns. Some irregularities toward the end are due to missing and faulty entries in the source PDFs.

### 3.2 Version 2: Cleaned and Verified

This version reflects a manually curated state of the dataset. Duplicate records were identified and removed through cross-verification with the official BPDB daily reports. Missing entries were retrieved directly from source documents to ensure data completeness. The dataset in this version contains consistent and validated daily records for national peak demand, total generation, temperature in Dhaka, and zone-wise demand, supply, and load.

**Fig. 2:**
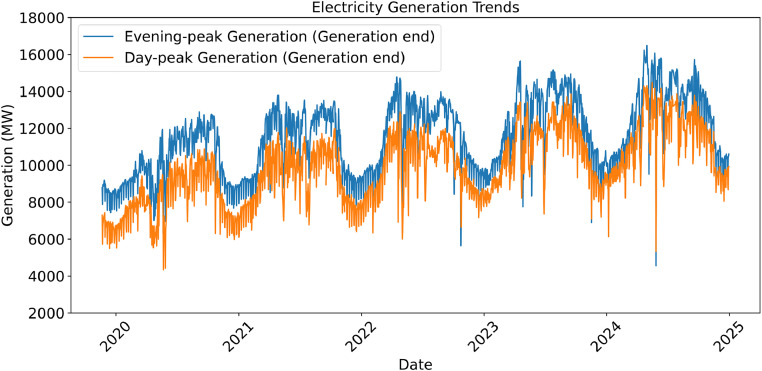
Daily Trends of Peak Electricity Generation (2019–2024) - Cleaned and Verified Data.

Fig. 2 represents the curated state of the dataset following manual validation. As seen in the figure, the dataset appears more complete, consistent, and structured after the cleaning process. Errors from duplicate and faulty entries have been resolved, enabling more reliable analysis.

### 3.3 Version 3: Holiday Feature Enrichment

To enhance contextual utility, in this version of the dataset, a new column is manually added to flag whether a given date corresponds to a holiday, based on the official calendar of Bangladesh. This includes national observances, religious holidays, and weekends. This calendar is published each year on the official website of the Government Press (Bangladesh Gazette) [3]. For dates marked as holidays, an additional column specifies the category of the holiday, offering enhanced contextual information for temporal analysis. The inclusion of holiday information can be useful, as electricity demand often exhibits distinct patterns on holidays compared to regular weekdays, thereby improving the accuracy of forecasting models and enabling better understanding of consumption behaviour.

### 3.4 Version 4: Interpolated and Structured

This refined version resolves all remaining missing and anomalous values using a combination of interpolation techniques and conditional averaging. It builds upon the structure of earlier versions while introducing two new columns: one for the year and another for the month. These additions facilitate more efficient temporal grouping and feature engineering for modeling tasks.

### 3.5 Version 5: Finalized for Modeling

Prepared specifically for ML and public release, this version applies final adjustments such as outlier correction and normalization of continuous variables (e.g., demand, supply, generation). While preserving the structure and enriched features from Version 4, this edition ensures each row contains a clean, complete, and model-ready daily record suitable for downstream analytics.

**Fig. 3:**
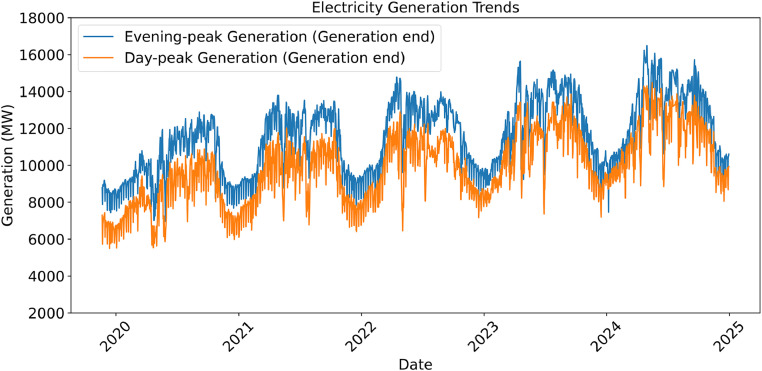
Daily Trends of Peak Electricity Generation (2019–2024) - Finalized Data.

Fig. 3 presents the daily evening and day peak electricity generation from 2019 to 2024, based on a cleaned and finalized dataset. Outliers and anomalies have been corrected to ensure each entry represents a reliable, complete, and analysis-ready record for modeling or interpretation.

**Fig. 4:**
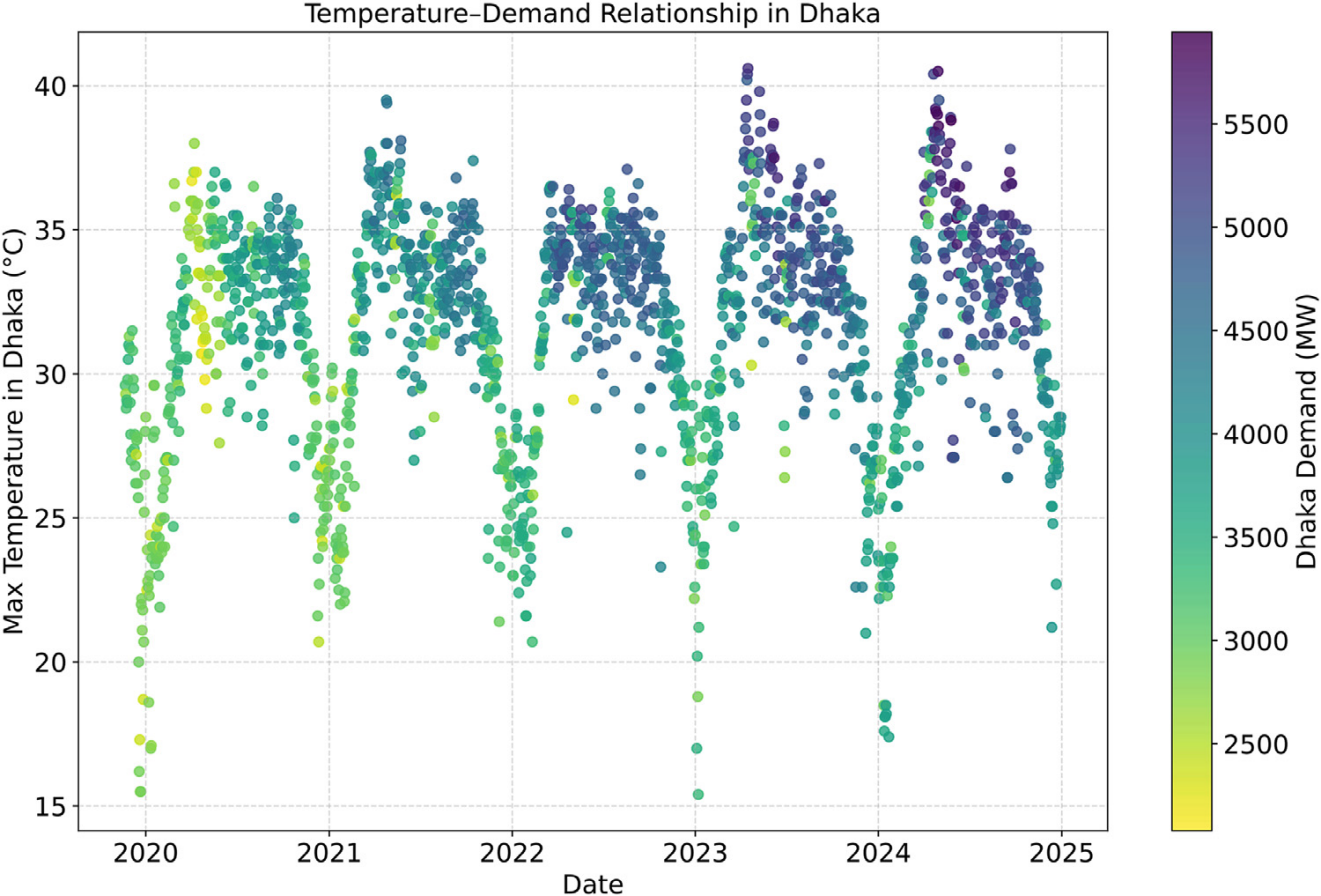
Max Temperature – Demand Relationship in Dhaka.

Fig. 4 visualizes the relationship between daily maximum temperature in Dhaka and electricity demand over time. Each point’s color reflects Dhaka’s power demand: warmer colors (yellow) represent lower demand, while cooler colors (purple) indicate higher demand. Seasonal temperature cycles are clearly visible, and a general trend of higher demand during hotter periods can be observed.

**Fig. 5:**
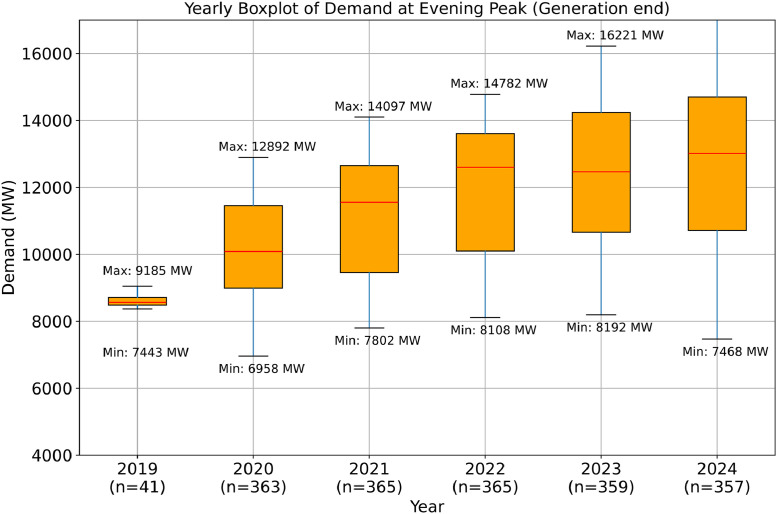
Yearly Distribution of Electricity Demand at Evening (Generation End).

Fig. 5 summarizes the yearly distribution of electricity demand at the evening peak from 2019 to 2024. The red lines indicate median values, while the upper and lower edges of each box represent the interquartile range, capturing the middle 50 % of daily demand values. Maximum and minimum demand levels are annotated, revealing a consistent upward trend in peak demand over time, with wider spread in later years due to higher fluctuations.

Table 1 provides a side-by-side comparison of the five dataset versions, highlighting column counts, new variables, and key processing steps.

**Table 1:** Comparison of dataset versions (V1–V5).

Version	No. of Columns	New Columns Added	Key Changes Compared to Previous Version
V1: Raw Extract	41	–	Direct extraction from BPDB PDFs; raw and unprocessed; inconsistent column names (e.g., “Actual data of”), duplicates, missing values, and zero entries all retained.
V2: Cleaned & Verified	41	Date (renamed from Actual data of)	Manual cleaning and verification; duplicate removal; standardized column names (Date, Demand, Generation, etc.); missing entries filled from original BPDB reports.
V3: Holiday Enrichment	43	Holiday name, Holiday_cat	Holiday contextual information added based on national calendar (weekends, religious, and national holidays); enables temporal demand analysis across holiday types.
V4: Interpolated & Structured	45	Year, Month	Interpolated missing/anomalous values; added 2 new columns (Year, Month) to facilitate temporal grouping and feature engineering; dataset now fully structured for modeling tasks.
V5: Finalized for Modeling	45	–	Outlier correction and normalization of continuous variables (demand, supply, generation); preserves enriched features from V3 and V4; model-ready dataset finalized.

Table 2 presents a comprehensive description of all variables included in the dataset. Each row represents daily observations and includes features such as electricity demand, temperature, generation levels, and regional breakdowns.

**Table 2:** Column-wise description of the dataset variables included in the final version (Version 5).

Column Name	Description
Date	Calendar date of the data entry (YYYY-MM-DD).
Day of the week	Name of the weekday (e.g., Sunday, Monday).
Year	Four-digit year associated with the entry.
Month	Numeric month (1–12) of the recorded data.
Max. Demand at eve. peak (Generation end)	Maximum national electricity demand recorded during the evening peak, measured at the generation side. For example, 9185 MW was recorded on a high-demand evening, indicating peak national load at generation.
Max. Demand at eve. peak (Sub-station end)	Maximum evening peak demand measured at the sub-station side, indicating delivery-side load. An entry of 8537 means 8537 MW was drawn at the distribution end during the evening peak.
Highest Generation (Generation end)	Maximum electricity generation achieved in a day. The highest daily output can reach values like 9185 MW on particularly demanding days.
Minimum Generation (Generation end)	Lowest electricity generation observed in a day. On days with reduced power usage, this might drop to levels like 5283.5 MW.
Day-peak Generation (Generation end)	Highest generation value during the daytime period. For example, the peak generation during the day reached 7433.9 MW.
Evening-peak Generation (Generation end)	Generation output specifically during the evening peak hours. For instance, 9051 MW may be generated during peak evening hours to meet demand.
Minimum Generation Forecast up to 8:00 hrs.	Forecasted minimum generation up to 8:00 AM, issued by BPDB. For example, if this column shows 4100, it means BPDB forecasted that generation could fall as low as 4100 MW by 8:00 AM on that day.
Maximum Temperature in Dhaka was	Daily maximum air temperature recorded in Dhaka. For instance, a value of 35.4 means the highest temperature recorded in Dhaka that day was 35.4 °C.
Gas/LF limitation	Reported generation shortfall due to gas or liquid fuel supply issues. An entry such as 1349 indicates that 1349 MW could not be generated due to fuel shortages.
Coal supply Limitation	Reported impact on generation due to coal availability constraints. A value of 450 indicates a generation deficit of 450 MW due to lack of coal.
Low water level in Kaptai lake	Indicator of hydro generation constraints due to low water level in Kaptai Lake. For example, if 217 MW is recorded, it indicates a 217 MW reduction in potential generation at the Kaptai hydro station caused by insufficient water levels.
Plants under shutdown/ maintenance	Number of generation plants reported offline due to maintenance or shutdown. A value of 6 indicates that six plants were not operational on that date.
[Region]_demand	Daily electricity demand values (in MW) for eight administrative divisions (Dhaka, Chattogram, Rajshahi, Khulna, Rangpur, Mymensingh, Sylhet, Barishal), plus Cumilla, is reported separately by BPDB. *For instance, if Dhaka demand is 2941, it means that Dhaka consumed 2941 MW of electricity that day.*
[Region]_supply	Electricity supply (in MW) allocated to the same nine regions listed above. *Example: A value of 2815 under Khulna supply indicates that 2815 MW was distributed to Khulna on that date.*
[Region]_load	Daily load shedding values (in MW) recorded across those nine regions. *For example, if Sylhet load is 26, this means Sylhet experienced 26 MW of unmet demand due to supply shortfall or system constraints.*
Holiday name	Name of the holiday (e.g., Eid-ul-Fitr, Victory Day) for the given date.
Holiday_cat	Categorization of the day: 0 = Normal day (Not a holiday), 1 = Single-day holiday, 2 = Multi-day holiday, 3 = Special occasion (e.g., Ramadan).

## 4 Experimental Design, Materials and Methods

### 4.1 Data Source and Context

BPDB maintains a publicly accessible digital archive that is updated daily [4]. For this study, a total of 1857 PDF reports were systematically collected from the official BPDB website using Python-based automation tools. Each report corresponds to a specific calendar date and presents structured energy statistics, typically in a tabular format on the second page. Relevant columns from the collected PDF reports were extracted using Python libraries, and the parsed data were then converted into a structured, machine-readable format suitable for time series analysis and deep learning-based forecasting.

### 4.2 Tools and Libraries Used

The data extraction and processing pipeline utilized a combination of Python libraries, each serving a specific role in web scraping, PDF parsing, data structuring, and cloud integration. A summary of the tools and their purposes is provided in Table 3.

**Table 3:** Python libraries and tools used in the data extraction and processing workflow.

Library/Tool	Purpose	References
requests	Fetches web content (HTML, PDFs) via HTTP GET requests.	[5]
BeautifulSoup	Parses HTML to extract and filter hyperlink tags.	[6]
urllib3	Suppresses SSL-related warnings from unverified sites.	[7]
re	Matches patterns in text using regular expressions.	[8]
os	Handles file paths, directory creation, and traversal.	[9]
pdfplumber	Extracts text from PDF pages while preserving relative layout and structure.	[10]
pandas	Cleans, structures, and exports tabular data in DataFrame and Excel-compatible formats.	[11]

### 4.3 Automated PDF Retrieval

A custom Python-based scraper was developed to download all relevant PDF reports from the BPDB archive. This process comprised multiple stages, integrating essential Python libraries and handling both static and dynamic web content. The scraping pipeline relied on multiple libraries: requests, BeautifulSoup, urllib3, re, and os. These tools worked together to extract PDF links embedded in paginated, browser-rendered HTML content hosted at: https://misc.bpdb.gov.bd/daily-generation-archive [4].

### 4.4 HTTP Requests and URL Handling

The requests library was used to send HTTP GET requests to each paginated URL within the archive. Each page (e.g., https://misc.bpdb.gov.bd/daily-generation-archive?page=X) contains hyperlinks to 10 daily PDF reports. The script looped through the archive pages using a page index (X), starting from 1 (the most recent) and continuing until all available reports within the desired date range were covered. As the number of pages in the archive varies over time, the script was designed to dynamically adjust the page index (X) during execution to ensure complete coverage of the target reporting period.

### 4.5 HTML Parsing and Link Filtering

The downloaded HTML content was parsed using BeautifulSoup, a Python library that facilitates parsing and searching HTML/XML trees. Filenames were filtered using regular expressions (re) to select only those matching either the pattern summary_*.pdf or \d{4}report.pdf.

### 4.6 PDF File Downloading

For each valid PDF link, the script invoked a custom download_file() function, which used requests to download the file in chunks (8 KB per chunk) and save it locally. This chunked download method was chosen to prevent memory overload during bulk downloads. Each file was saved using its original filename into a specified directory path.

### 4.7 PDF Text Extraction and Parsing

Once the reports were downloaded, the next stage involved extracting structured data from PDF documents. This was accomplished using pdfplumber, a Python library specialized in extracting text from PDF pages while preserving relative layout. Each report was opened using pdfplumber.open(), and the content was extracted from page 2 (index 1). The extracted text was then parsed using Python’s re (regular expressions) module to isolate relevant numeric and categorical values spanning demand, generation, temperature, fuel limitations, and regional breakdowns.

### 4.8 Data Structuring and Excel Integration

After parsing, all dictionaries were converted into a pandas DataFrame. To maintain consistency across records, a fixed list of expected column names was defined. Each extracted data dictionary was validated against this list, and missing fields were filled with None. All records were cumulatively stored in a master .xlsx file (data.xlsx) using pandas.to_excel().

If the file already existed, new entries were appended to the existing DataFrame to ensure seamless updates. A separate errors.txt file was generated to record any PDF processing failures, such as unreadable pages or missing expected structure.

## 5 Data preprocessing and feature engineering

### 5.1 Missing Value Handling

After extraction, the dataset initially contained a considerable number of missing values due to faulty characters and inconsistent formatting in several of the source PDFs. These issues prevented pdfplumber from accurately detecting numeric values, especially where characters were partially broken or replaced by non-standard symbols. For example, *Khulna_demand, Khulna_supply*, and *Khulna_load* each had 61 missing entries due to repeated formatting issues in that region’s reports. Similar problems were observed in other fields, including Max*. Demand at eve. peak (Sub-station end), Highest Generation (Generation end), Minimum Generation (Generation end), Day-peak Generation (Generation end), Evening-peak Generation (Generation end), Minimum Generation Forecast up to 8:00* hrs*., Maximum Temperature in Dhaka was, Gas/LF limitation, Coal supply Limitation, Low water level in Kaptai lake*, and *Plants under shut down/ maintenance*, each with 1 to 5 missing values. The column Max*. Demand at eve. peak (Generation end)* also contained 30 zero entries, which were treated as invalid due to their implausibility. Region-level electricity statistics were also affected: *Dhaka_demand/supply/load* fields had 9 missing entries each; *Chattogram_demand/supply/load* fields had 7 each; *Rajshahi_demand/supply/load* fields had 7 each; *Mymensingh, Sylhet, Barishal*, and *Rangpur_demand/supply/load* fields had 1 each; and *Cumilla_demand/supply/load* fields had 4 each.

These faulty entries were manually reviewed and corrected using the original PDF documents. After this manual cleaning and correction process, the dataset still exhibited 40 unresolved missing entries across the following columns: Max*. Demand at eve. peak (Generation end)* (30), *Minimum Generation Forecast up to 8:00* hrs*.* (1), *Maximum Temperature in Dhaka* (5), *Gas/LF limitation* (1), *Coal supply Limitation* (1), *Low water level in Kaptai lake* (1), and *Plants under shut down/ maintenance* (1).

The remaining missing values were primarily addressed using interpolation within date sequences. Internal gaps were filled using both forward and backward directions. For Max*. Demand at eve. peak (Generation end)*, the zero values were imputed using the average of two components: (i) an interpolated estimate from the ten preceding and ten succeeding non-zero values, and (ii) the corresponding substation-end peak demand value. This approach ensured plausible and context-aware replacements. Additionally, any corrupted PDFs that resulted in fully missing or malformed rows were recorded into an error log and manually inspected to determine if recovery was feasible.

### 5.2 Holiday Feature Construction

Each entry in the dataset was cross-referenced with a curated list of national and religious holidays in Bangladesh. A new categorical variable was introduced to classify each day into one of four categories: regular working days (0), single-day holidays such as Independence Day, Victory Day, or International Mother Language Day (1), continuous multi-day holidays such as Puja or Eid (2), and special occasions like Ramadan, during which electricity consumption tends to be higher than on regular days (3). This feature was instrumental in capturing demand variability linked to calendar effects and socio-cultural factors.

### 5.3 Outlier Detection and Correction

Outliers were corrected using a hybrid approach combining rolling median smoothing with strict deviation thresholds. A 5-day centered rolling median was computed for each critical column, such as generation, demand, and supply. Any value deviating >30 % from this local median was flagged as an outlier. These flagged values were then replaced with the corresponding rolling median, with forward- and backward-filling applied to address edge cases. This method was implemented across all key numerical fields, including zone-wise demand and supply, and provided robust smoothing while preserving genuine temporal variability.

**Fig. 6:**
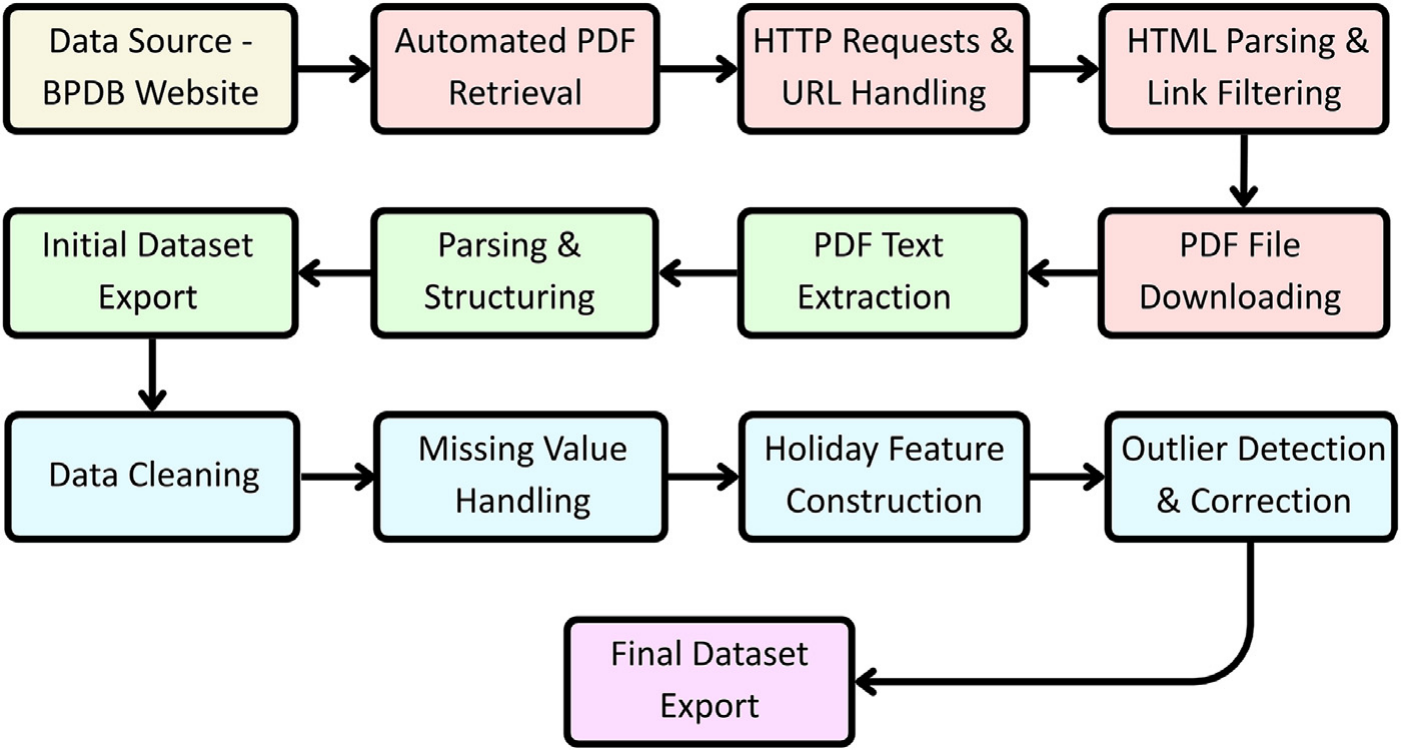
Flowchart of the automated data extraction and preprocessing pipeline.

Fig. 6 illustrates the automated workflow, beginning with raw data extraction from BPDB daily reports, followed by systematic preprocessing steps, and culminating in the generation of a structured, reproducible dataset in Excel format.

To ensure reproducibility and reuse, the entire Python-based pipeline is available in a dedicated GitHub repository: https://github.com/codebyrama/Bangladesh-Power-Data-Pipeline [12]. The repository is organized into clear, modular Jupyter notebooks. The first notebook, **01_bpdb_pdf_scraping.ipynb**, automates the process of collecting daily PDF reports from the BPDB archive[4] and converting them into structured Excel files. The second notebook, **02_data_preprocessing.ipynb**, takes these raw extractions through systematic cleaning, validation, and feature engineering. Together, they allow any researcher to recreate the dataset for a chosen date range directly from the BPDB website and to replicate the exact preprocessing steps used in this study. By following this structure, users can trace the workflow step by step, adapt individual modules, or reproduce the full pipeline seamlessly.

## Limitations

One limitation of the dataset is the absence of hourly electricity data. Although the initial objective was to compile a higher-resolution dataset with hourly granularity, such data is not publicly available in a suitable or accessible format from official sources. As a result, the dataset is limited to daily records, which may restrict certain types of fine-grained temporal analysis or short-term load forecasting applications.

## Ethics Statement

The authors confirm that they have read and followed the ethical requirements for publication in Data in Brief. This work does not involve human subjects, animal experiments, or any data collected from social media platforms. All data were programmatically collected from publicly available government reports in accordance with their usage terms. The dataset contains no personal or sensitive information and complies with ethical standards for transparency, reproducibility, and responsible data sharing in computational research.

## Credit Author Statement

**Md. Ikrama Hossain:** Writing – original draft, Writing – review & editing, Software, Methodology, Investigation, Data curation, Conceptualization. **Tasnia Nafs:** Writing – original draft, Writing – review & editing, Software, Methodology, Investigation, Data curation, Conceptualization. **Sakif Yeaser:** Writing – original draft, Writing – review & editing, Software, Methodology, Investigation, Data curation, Conceptualization. **Asif Newaz:** Supervision, Writing – review & editing.

## Data Availability

Mendeley DataStructured Dataset of Daily Electricity Demand, Generation, Load Shedding, and Supply Constraints in Bangladesh (2019–2024). (Original data).
